# Audit of Non-Pharmacological and Rapid Tranquilisation Practices in Managing Distress Among Older Adults: A Comparative Study in Inverness Hospitals

**DOI:** 10.1192/bjo.2024.594

**Published:** 2024-08-01

**Authors:** Praveen Kumar, Adam Wild, Fiona Howells, Tharini Kumar, Phoebe Williams

**Affiliations:** 1 New Craig's Psychiatric Hospital, Inverness, United Kingdom; 2 Raigmore Hospital, Inverness, United Kingdom; 3 South Tipperary General Hospital, Clonmel, Ireland; 4 University of Aberdeen, Aberdeen, United Kingdom

## Abstract

**Aims:**

This audit evaluates the adherence of nursing and medical staff to local protocols for managing distress in older adults (aged >65 years) using non-pharmacological approaches and rapid tranquilisation (RT) in a psychiatric hospital's dementia ward, an acute medical unit, and a geriatric ward in a general hospital. We hypothesize that operational differences between these wards significantly influence the management of older adult patients.

**Methods:**

Conducted from September 17 to October 8, 2022, in hospitals in Inverness, Scotland, this study reviewed 322 case notes and drug charts from patients who underwent RT in three wards: the Old Age Psychiatric Ward, Acute Medical Unit (AMU), and Geriatric Ward. Focus groups and informal discussions with ward nurses and junior doctors were organized to understand their perspectives on handling distress in dementia patients, with an emphasis on de-escalation techniques.

Data focused on key parameters:

- Patient Diagnosis and Legal Status.
- Administration Details: including initiation time, de-escalation techniques, consultation with senior doctors, and details of drugs administered (route, drug, and dosage).

**Results:**

Staff nurses in all wards prioritized non-pharmacological de-escalation techniques, such as recognizing early signs of agitation, employing distraction and calming tactics, and acknowledging the importance of personal space, even in the face of staffing challenges and high patient loads. These measures were consistently employed prior to considering RT, adhering to the local protocol. Physical restraint was employed only in scenarios where there was a risk to the patient or others, executed by personnel trained in managing violence and aggression.

Conversations with junior doctors, particularly in the AMU, revealed a limited understanding of the RT protocol, suggesting a need for enhanced training and awareness. Overall, the study indicates that while RT is regarded as a last resort after the failure of psychological and behavioral approaches, there is a clear necessity for further education and training to ensure the safe and effective administration of RT.

**Conclusion:**

This audit demonstrates that despite the varying environments and pressures in the three wards, adherence to the local protocol for managing distress in older adults is largely effective, with a strong preference for non-pharmacological methods. The findings highlight the need for ongoing education and reinforcement of RT protocols, particularly among junior doctors, to ensure patient safety and adherence to best practices. The results suggest that with proper support and training, the use of RT can be a carefully controlled and beneficial tool in managing distress in older adult patients.

